# The HDL mimetic CER‐001 remodels plasma lipoproteins and reduces kidney lipid deposits in inherited lecithin:cholesterol acyltransferase deficiency

**DOI:** 10.1111/joim.13404

**Published:** 2021-11-11

**Authors:** Chiara Pavanello, Marta Turri, Arianna Strazzella, Patrizia Tulissi, Stefano Pizzolitto, Giovanna De Maglio, Riccardo Nappi, Laura Calabresi, Giuliano Boscutti

**Affiliations:** ^1^ Dipartimento di Scienze Farmacologiche e Biomolecolari Centro E. Grossi Paoletti Università degli Studi di Milano Milan Italy; ^2^ Unit of Nephrology, Dialysis and Renal Transplantation, S. Maria della Misericordia Hospital Azienda Sanitaria Universitaria Friuli Centrale (ASUFC) Udine Italy; ^3^ Unit of Pathology, S. Maria della Misericordia Hospital Azienda Sanitaria Universitaria Friuli Centrale (ASUFC) Udine Italy

**Keywords:** chronic kidney disease, lipids, familial LCAT deficiency, HDL mimetic, CER‐001

## Abstract

**Background:**

Kidney failure is the major cause of morbidity and mortality in familial lecithin:cholesterol acyltransferase deficiency (FLD), a rare inherited lipid disorder with no cure. Lipoprotein X (LpX), an abnormal lipoprotein, is primarily accountable for nephrotoxicity.

**Methods:**

CER‐001 was tested in an FLD patient with dramatic kidney disease for 12 weeks.

**Results:**

Infusions of CER‐001 normalized the lipoprotein profile, with a disappearance of the abnormal LpX in favour of normal‐sized LDL. The worsening of kidney function was slowed by the treatment, and kidney biopsy showed a slight reduction of lipid deposits and a stabilization of the disease. In vitro experiments demonstrate that CER‐001 progressively reverts lipid accumulation in podocytes by a dual effect: remodelling plasma lipoproteins and removing LpX‐induced lipid deposit.

**Conclusion:**

This study demonstrates that CER‐001 may represent a therapeutic option in FLD patients. It also has the potential to be beneficial in other renal diseases characterized by kidney lipid deposits.

## Introduction

Familial lecithin:cholesterol acyltransferase (LCAT) deficiency (FLD, OMIM#245900) is a rare recessive disorder of HDL metabolism, due to loss‐of‐function mutations in the *LCAT* gene [1]. Glomerulosclerosis is the major cause of morbidity and mortality in FLD patients, and kidney failure occurs in the 4th–5th decade of life [2]. FLD carriers display very low HDL‐cholesterol levels and severe defects in plasma cholesterol esterification, resulting in the virtual absence of circulating cholesteryl esters [1]. Excess of unesterified cholesterol cannot be sustained by plasma lipoproteins and forms an abnormal lipoprotein called lipoprotein X (LpX), which deposits in the glomerulus, inducing kidney damage [3]. Indeed, unesterified cholesterol is the only biochemical parameter so far able to identify patients with rapid deterioration of kidney function [2].

Kidney transplantation represents an option in severe cases of end‐stage renal disease; however, the systemic disease leads to a median time to a second recurrence after transplantation of 10 years [2]. Enzyme replacement therapy with recombinant LCAT [4, 5] and LCAT activators [6, 7] represent therapeutic options, but clinical development is lagging. CER‐001, an HDL mimetic, has been previously tested for atherosclerosis regression, by virtue of its capacity to rapidly mobilize large amounts of cholesterol from peripheral cells [8]. Recently, our group reported that CER‐001 ameliorated kidney disease in a mouse model of LCAT deficiency, by limiting the glomerular accumulation of lipid droplets and limiting albuminuria and podocyte damage [9]. On this basis, CER‐001 was tested in an FLD patient presenting with extremely fast recurrence of kidney disease, with the aim of delaying disease progression.

## Methods

### Study medication

CER‐001 is a negatively charged HDL mimetic, containing recombinant human apolipoprotein A‐I (apoA‐I) bound to sphingomyelin and dipalmitoyl phosphoglycerol [8], able to promote cholesterol mobilization [10]. CER‐001 infusion was tested through a compassionate program, approved by the local ethical committee (Approval nr. CEUR‐2020‐EAP‐012‐ASUFC) in cooperation with Abionyx Pharma, which provided the study medication. CER‐001 was infused at a dose of 10 mg/kg 3 times per week for 3 weeks, followed by 10 mg/kg 2 times per week for 3 weeks, and then 10 mg/kg once per week for 6 weeks.

### Biochemical analyses

Fasting blood samples were collected at all visits and at the end of treatment (EOT), immediately before CER‐001 administration, and plasma was separated by low‐speed centrifugation at 4°C. Details about biochemical determinations are described in the supporting information.

### Kidney biopsy

Standard processing of kidney biopsy samples included light microscopy, immunofluorescence and transmission electron microscopy. Further details are described in the supporting information.

### Cell studies

In vitro experiments were performed using immortalized human podocytes [11]. A detailed description of cell studies is reported in the supporting information.

### Statistical analysis

Data are expressed as mean ± SD. Statistical analysis for cell studies was carried out using SigmaPlot 12.5 (SystatSoftware Inc., San Jose, CA, USA). Normality was tested using the Shapiro–Wilk test. Normally distributed data were evaluated using a 2‐tailed paired Student's *t*‐test or a one‐way ANOVA for repeated measures as appropriate. Bonferroni's post‐hoc test was applied for multiple comparisons. A threshold level of significance was set at a *p*‐value <0.05.

## Results

### Characteristics of the study patient

**Fig. 1:**
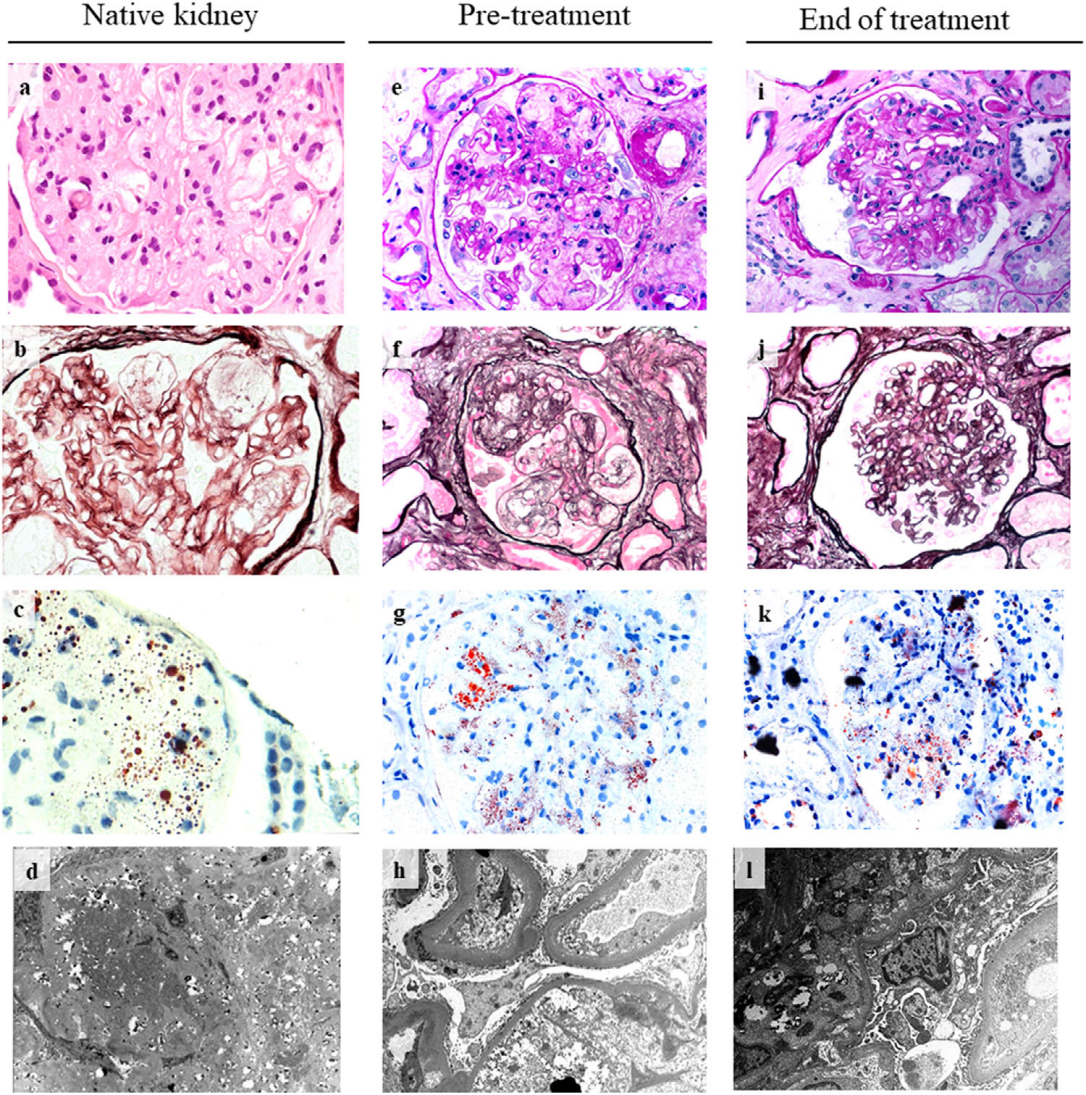
Biopsy of native kidney (a–d), transplanted kidney before (e–h) and after treatment (i–l). (a) Representative image of biopsy of native kidney, stained with hematoxylin/eosin (40x). (b) Periodic Schiff‐Methenamine Silver (PASM) stain (40x). (c) Oil red O stain (40x). (d) Electron micrograph (9000x). (e,i) Representative image of biopsy of transplanted kidney before and after treatment with CER‐001, stained with Periodic acid–Schiff (PAS)(40x). (f,j) PASM stain (40x). (g,k) Oil red O stain (40x). (h, l) Electron micrograph (9000x and 11000x).

**Fig. 2:**
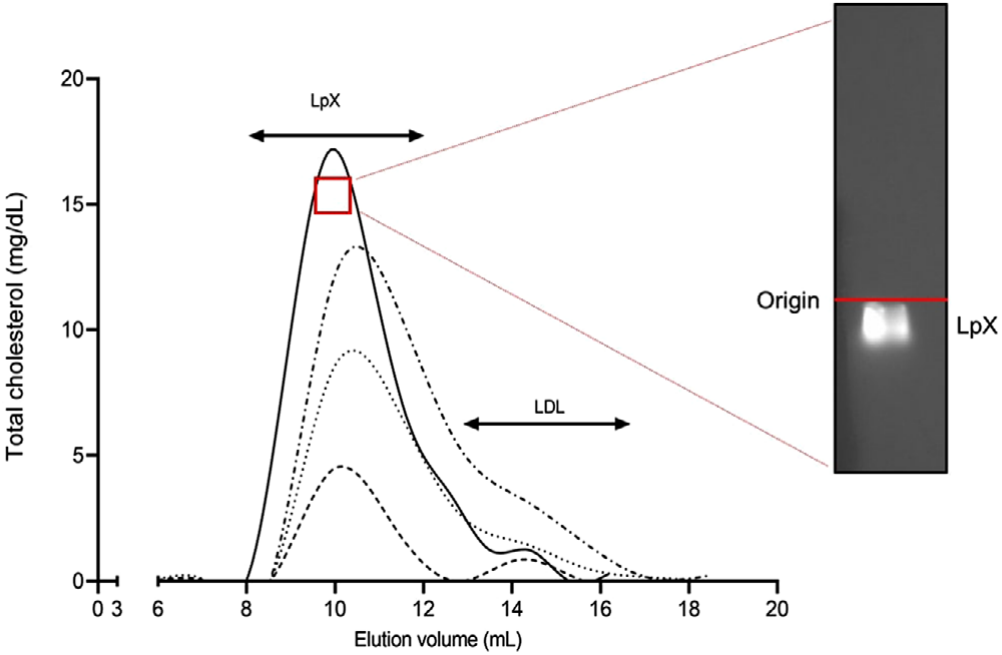
Separation of the 1.020–1.063 g/ml plasma fraction by fast protein liquid chromatography (FPLC). The 1.020–1.063 g/ml density fraction of plasma fraction, corresponding to lipoprotein X and LDL, was separated by FPLC and analysed for lipid content at baseline (solid line), at week‐3 (dash‐dotted line), week‐6 (dotted line) and the end of treatment (EOT) (dashed line). Insert depicts agarose gel of patient's plasma obtained at baseline stained with filipin, which specifically binds to unesterified cholesterol, to reveal the presence of plasma lipoprotein X. FPLC denotes fast performance liquid chromatography, LpX lipoprotein X.

The patient is a 49‐year‐old Italian male with genetically proven FLD, due to homozygous Thr274Ile mutation in LCAT [12]. At first referral at the nephrology department at the age of 27, FLD was confirmed by kidney biopsy, which revealed mesangial expansion and irregular thickening of the glomerular capillary walls, together with lipid droplets deposition (Fig. 1a–d). The patient showed an extremely fast recurrence of renal disease, which required three kidney transplantations within 20 years. In 9 months from the last renal graft, his kidney function quickly dropped by half. The overabundance of plasma unesterified cholesterol (Table S1) in this FLD patient could partially explain his severe clinical condition [2]. Indeed, a large amount of LpX was detected in the patient's plasma on the agarose gel stained with Filipin (Fig. 2, inset).

Because of the very fast recurrence of the LCAT nephropathy in the graft, treatment with CER‐001 was started through a compassionate programme. The patient was already receiving immunosuppressive (methylprednisolone, mycophenolate mofetil, and tacrolimus) and anti‐hypertensive (carvedilol, amlodipine, and ramipril) therapy and epoetin. All drugs were continued during CER‐001 treatment. The patient had never taken lipid‐lowering drugs.

Kidney biopsy (pre‐treatment) was performed (Fig. 1e–h). Electron microscopy showed reappearance of glomerular lipid deposition consistent with recurrence of LCAT deficiency (Fig. 1h, lower), whilst antibody‐mediated graft rejection in a hyperimmunized patient was excluded by anti‐human leukocyte antigens analysis (data not shown). Most analysed glomeruli (15 out of 23) were globally or sub‐globally sclerosed. The remaining glomeruli showed minimal alterations or amorphous deposits in the mesangial and capillary cells. Segmental glomerular sclerosis was observed with irregular thickening of the glomerular capillary walls and vacuolization of the glomerular basement membrane due to intramembranous lipid deposits, resulting in a typical foamy appearance. The tubulointerstitial space showed extensive fibrosis, with chronic inflammatory infiltrates and tubular atrophy, and moderate to severe thickening of the tubular vascular wall. Oil red O staining revealed glomerular deposition of lipids. Immunofluorescence was slightly positive for C3 and C1q and for IgM, likely unspecific, but was negative for C4d (Fig. 1f–h).

### CER‐001 reduces kidney lipid deposits

After 12 weeks of treatment, kidney biopsy was repeated. Histological analysis proved a modest reduction of the glomerular lipid deposition, despite the presence of fibrosis and atrophy, suggesting a partial amelioration of kidney disease and an overall stabilization (Fig. 1i–l). In addition, immunofluorescence was negative for complement deposition (Fig. 1i–l), which has been previously reported as a marker of fast disease progression [13]. Although no striking improvement could be appreciated with CER‐001 treatment, the worsening of kidney function was somehow slowed by the treatment. In fact, whilst estimated glomerular filtration rate (eGFR) decreased from 38.3 to 18.3 ml/min/1.73 m^2^ in the 12 months before the beginning of CER‐001 treatment (1.65 ml/min/1.73 m^2^ per month), eGFR declined to 13 ml/min/1.73 m^2^ in 3.4 months of treatment (1.54 ml/min/1.73 m^2^ per month) (Fig. S1). Both albumin‐to‐creatinine and protein‐to‐creatinine ratios increased in the first 3 weeks of treatment, then dropped in the following weeks (Table S1). The same trend was observed for proteinuria (Table S1). The effect of a more prolonged administration could not be evaluated due to CER‐001 unavailability but would have likely led to a better result as shown in the French case [14]. eGFR decline resumed and haemodialysis was consequently restarted. Treatment was well‐tolerated, and no adverse events were observed.

### CER‐001 remodels patient's plasma lipoproteins reducing LpX

The patient's biochemical characteristics at baseline and after CER‐001 treatment are reported in Table S1. As expected, the patient presented with dramatically low HDL‐cholesterol, apoA‐I and apoA‐II levels. Infusions of CER‐001 did not substantially change lipid levels (Table S1). However, a 71% reduction in triglycerides was observed after 3 weeks of CER‐001 at the highest dose regimen (3x week) as seen in the mouse model [9]. After 3 weeks of treatment, unesterified cholesterol transiently increased by 25%, likely due to cholesterol mobilization from kidney cells promoted by CER‐001 [8], then slowly returned to baseline levels at the EOT (Table S1). To evaluate the effect of CER‐001 on circulating LpX, the ultracentrifugally obtained 1.020–1.063 g/ml fraction, containing LDL and LpX, was resolved using fast protein liquid chromatography. Cholesterol profiles demonstrated that LpX was the most prominent lipoprotein at baseline (Fig. 2), confirmed by agarose gel followed by filipin staining (Fig. 2, inset). Treatment with CER‐001 led to a normalization of the lipoprotein profile, with the disappearance of the abnormal LpX in favour of normal‐sized LDL (Fig. 2).

As already described in the CARAT trial [15], HDL‐cholesterol and apoA‐I levels did not change with CER‐001 Atherosclerosis Regression ACS Trial infusions (Table S1).

### Beneficial effect of CER‐001 is mediated by lipoprotein remodelling and direct cholesterol removal from kidney cells

**Fig. 3:**
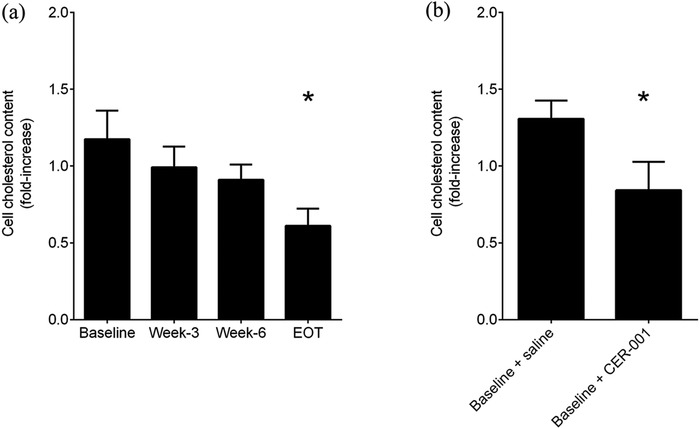
CER‐001 limits cholesterol deposition in cultured podocytes through lipoprotein remodelling and by direct cholesterol efflux. (a) Podocytes were starved in a serum‐free medium for 2 h and then incubated for 48 h with the patient's plasma collected at different time points. Cells were washed and lysed, and intracellular cholesterol was measured and normalized to protein concentration. Data are expressed as a fold change in cholesterol content in treated versus untreated cells. Data are mean ± SD of three different experiments and were analysed by one‐way ANOVA for repeated measures followed by Bonferroni's post hoc test. *P‐value versus baseline = 0.009. EOT denotes the end of treatment. (b) Same as in (a) but podocytes were treated with baseline patient's plasma for 48 h and then treated for 4 h with saline solution or CER‐001. Data are mean ± SD of three different experiments and were analysed by paired Student's t‐test. *P‐value versus saline = 0.001.

With the aim of clarifying the mechanism(s) of the beneficial effect observed with CER‐001, we carried out in vitro experiments with podocytes, the main cells involved in FLD‐induced kidney damage. Incubation of cells with plasma collected from the patient at baseline produced an increase in cell cholesterol content (Fig. 3a), in accordance with the high content of circulating LpX (Fig. 2). Incubation of cells with the patient's plasma collected after CER‐001 treatment progressively led to less lipid accumulation in kidney cells (Fig. 3a), confirming that the drug‐induced remodelling of plasma lipoproteins (Fig. 2) is responsible for the reduced cholesterol deposit in cells. Indeed, at EOT, when the lipoprotein remodelling was most evident, the deposition of cholesterol in podocytes was significantly reduced (Fig. 3a). These results were confirmed by the incubation of podocytes with the lipoprotein fraction corresponding to LpX‐LDL, which recapitulated what was observed with whole plasma (Fig. S2). Finally, to assess the direct effect of CER‐001 on LpX‐induced lipid deposit, podocytes were preincubated with the patient's plasma collected at baseline and then exposed to CER‐001 at a concentration resembling the one in vivo. As shown in Figure 3b, CER‐001 rescued nephrotoxic cholesterol deposition, consistent with what was observed in the patient's biopsy.

## Discussion

The present report confirms the beneficial effects on the kidney of the HDL mimetic CER‐001 previously observed in an experimental model [9] and in a single case of LCAT deficiency [14], and provides novel insights into the mechanisms of kidney function stabilization exerted by the drug.

Kidney disease is the primary cause of morbidity and mortality in FLD patients [1], and kidney transplantation is unavoidable in the majority of cases. However, kidney transplantation is not corrective, and as for other systemic disorders characterized by kidney implications, it increases life expectancy, but graft survival is limited. At present, the major goal of therapeutic intervention in FLD remains the delay of kidney failure. Renoprotective agents can be used; however, their efficacy is limited [15, 16].

Here we report an extremely rapid recurrence of kidney disease in an FLD patient in his 40s, who underwent three kidney transplantations. He presented with an unusually rapid decline of kidney function within 1 year from the last transplantation, which suggests an exceptionally short graft survival. The patient was in chronic treatment with ramipril and other antihypertensive drugs; however, this was not enough to slow the deterioration of kidney function. In the absence of curative therapies, we hypothesized that the removal of kidney‐deposited cholesterol by CER‐001 could stabilize kidney disease.

Consistent with what was observed in the mouse model [9] and in a French FLD patient [14], CER‐001 infusion reduced lipid deposits in the glomeruli in our patient. As expected, this effect was not elicited by changes in lipid levels, which remained largely stable, but rather by a normalization of the lipoprotein profile induced by CER‐001. Indeed, LpX, which was the most prominent lipoprotein at baseline, partially disappeared in favour of normally sized LDL after treatment. The remodelling of plasma lipoproteins occurred slowly, and it reached a partial normalization at the EOT, thus suggesting a progressive process of LpX‐disassembling mediated by the infused HDL, which likely acts as shuttle/sink of unesterified cholesterol and phospholipids. In line with the drug‐induced lipoprotein normalization, in vitro experiments showed that whilst the patient's plasma at baseline induced cholesterol deposition in podocytes, plasma collected during CER‐001 treatment became progressively less prone to accumulate cholesterol in cells.

Besides accepting cholesterol from LpX and remodelling lipoproteins, CER‐001 could exert its positive effect by directly effluxing cholesterol from kidney cells, as it does in vascular cells [8]. This hypothesis was confirmed by in vitro experiments showing that CER‐001 was able to reduce intracellular cholesterol content in cultured podocytes loaded with the patient's plasma collected at baseline, and it is supported by the transient increase in unesterified cholesterol observed during treatment.

Overall, the present study confirms the hypothesis that the cholesterol removal capacity of CER‐001 can reduce the burden to the kidney of cholesterol deposition in FLD. The beneficial effect is mediated by a dual action of CER‐001, which directly effluxes cholesterol from podocytes, but also induces normalization of plasma lipoproteins, thus reducing lipid deposit in the kidney.

Despite the beneficial effects on plasma lipoproteins, CER‐001 infusion had very little effect on kidney disease in our case, likely because of the extremely compromised kidney status of the patient and the quite short duration of treatment. Indeed, the slower decline of kidney function together with the reduced lipid deposition in the glomeruli and the promising reduction in plasma LpX at the EOT suggest that better results could be achieved with a more prolonged treatment, as also suggested by the report by S. Faguer et al [14].

## Conclusions

FLD is a rare dyslipidaemia with severe kidney complications, presently with no cure. CER‐001 may represent a therapeutic option, with the objective of delaying kidney disease. Moreover, its use in combination with enzyme replacement therapy would represent a curative therapy for FLD patients. Importantly, CER‐001 has the potential to be tested in more common kidney diseases characterized by kidney lipid deposits.

## Conflict of interest

Laura Calabresi received research grants from Abionyx Pharma.

## Supporting information



Supporting Information
