# Supplementary material for: The HDL mimetic CER‐001 remodels plasma lipoproteins and reduces kidney lipid deposits in inherited lecithin:cholesterol acyltransferase deficiency
Source: J Intern Med. 2021 Nov 11;291(3):364–70. doi: 10.1111/joim.13404 (PMC9299003; doi:10.1111/joim.13404)
Supplement: Supplementary file 1 — Supporting Information [file JOIM-291-364-s001.pdf]

# Supporting information

## The HDL mimetic CER-001 remodels plasma lipoproteins and reduces kidney lipid deposits in inherited LCAT deficiency

*Running headline*: CER-001 in familial LCAT deficiency

C. Pavanello[1], M. Turri[1], A. Strazzella[1], P. Tulissi[2], S. Pizzolitto[3], G. De Maglio[3], R. Nappi[2], L. Calabresi[1] & G. Boscutti[2].

*From the [1]Centro E. Grossi Paoletti, Dipartimento di Scienze Farmacologiche e Biomolecolari, Università degli Studi di Milano, Milan, Italy; [2]Nephrology, Dialysis and Renal Transplantation Unit, S. Maria della Misericordia Hospital, Azienda Sanitaria Universitaria Friuli Centrale (ASUFC), Udine, Italy; [3]Pathology Unit, S. Maria della Misericordia Hospital, Azienda Sanitaria Universitaria Friuli Centrale (ASUFC), Udine, Italy.*

*Correspondence*: Laura Calabresi, PhD, Centro E. Grossi Paoletti, Dipartimento di Scienze Farmacologiche e Biomolecolari, Università degli Studi di Milano, Via Balzaretti 19, 20133 Milan, Italy. Phone: +39 0250319906 E-mail: laura.calabresi@unimi.it

## Methods

*Biochemical analyses*

A complete lipid-lipoprotein profile (total cholesterol, HDL-cholesterol, triglycerides, apolipoprotein A-I (apoA-I), apoA-II and apoB) was determined using a Roche Integra c311 analyzer. LDL-cholesterol was calculated by the Friedewald's formula. Plasma unesterified cholesterol and phospholipids were determined by standard enzymatic techniques. Since LpX has a similar density to LDL and a similar size to VLDL, to examine its presence in plasma at each timepoint, the 1.020–1.063 g/mL lipoprotein fraction was first separated by sequential ultracentrifugation and subsequently analyzed by FPLC using a Superose 6 HR 10/30 column (GE Healthcare,UK) [1]. Presence of LpX at baseline was additionally confirmed in fresh plasma by electrophoresis using Sebia agarose gels, followed by Filipin staining of unesterified cholesterol. Fluorescent spot corresponding to LpX on the gel was monitored by ChemiDoc (BioRad,Hercules,CA,USA) [2].

Estimated glomerular filtration rate (eGFR) was calculated using the 2009 CKD-EPI creatinine equation [3].

## Kidney biopsy

Tissue for light microscopy were formalin-fixed, paraffin-embedded. The successively obtained 3-µm-thick sections were stained with hematoxylin and eosin (H&E), periodic acid-Shiff (PAS), Masson's trichrome and Jones methenamine silver (PASM). For immunofluorescence 4-µm cryostat sections were stained with polyclonal fluorescein isothiocyanate-conjugated antibodies to IgG, IgM, IgA, C1q, C3, C4, fibrinogen, k, λ, C4d. Oil red O was yet used in fresh frozen tissue for the detection of lipoprotein droplets. Specimens for transmission electron microscopy study were fixed in 2% buffered

glutaraldehyde, postfixed in osmium tetroxide and embedded in propylene. Thin sections were stained with uranyl acetate and lead citrate.

**Cell studies**

Cell studies were performed using immortalized human podocytes maintained in Dulbecco's modified Eagle’s medium containing 1 g/L of glucose supplemented with 10% fetal bovine serum, 1% L-Glutamine, 1% antibiotics. 100’000 cells per well were seeded. To evaluate the effect of drug-induced lipoprotein remodeling on intracellular cholesterol content, podocytes were starved in serum-free medium for 2 h and then incubated for 48 h with 2% v/v of plasma collected at different time points. In a second set of experiments cells were incubated the ultracentrifugally obtained 1.020-1.063 g/mL lipoprotein fractions obtained at the same time points. In a third set of experiments, to assess the direct effect of CER-001 on intracellular cholesterol content, podocytes were pretreated with baseline patient’s plasma for 48 hours and then incubated for 4 h with CER-001 saline at a concentration resembling the dose of 10 mg/kg (0.15 mg/mL of protein) or with the same volume of saline solution. In all experiments, cells were then washed with PBS and lysed overnight with 1% sodium cholate and 10 U/mL DNase. Intracellular cholesterol content was assessed by fluorescence using the Amplex Red Cholesterol Assay kit (Sigma Aldrich) and the results were normalized by protein concentration in the total cell lysate, measured according to Lowry method. Change in cholesterol content was expressed as fold change versus non treated cells. All experiments were performed in triplicate with the same plasma and lipoprotein preparation.

## References


1 Hanna EV, Simonelli S, Chamney S, Ossoli A, Mullan RN. Paradoxical fall in proteinuria during pregnancy in an LCAT-deficient patient-A case report. *J Clin Lipidol*. 2018; **12**:1151-1156.

2 Ossoli A, Neufeld EB, Thacker SG, et al. Lipoprotein X Causes Renal Disease in LCAT Deficiency. *PLoS One*. 2016;**11**:e0150083.

3 Levey AS, Stevens LA, Schmid CH, et al. A new equation to estimate glomerular filtration rate. *Ann Intern Med*. 2009;**150**:604-12.

**Table S1. Effect of CER-001 on blood biochemical parameters.**

| | Baseline | Week-3 | Week-6 | EOT |
|---|---|---|---|---|
| CER-001 dose frequency | | 3xWeek | 2xWeek | 1xWeek |
| *Lipids and lipoproteins* | | | | |
| Total cholesterol (mg/dL) | 118 | 120 | 108 | 93 |
| Unesterified cholesterol (mg/dL) | 95 | 119 | 98 | 86 |
| Unesterified/total cholesterol | 0.80 | 0.99 | 0.90 | 0.93 |
| Triglycerides (mg/dL) | 235 | 69 | 131 | 188 |
| LDL-cholesterol (mg/dL) | 54 | 90 | 65 | 41 |
| HDL-cholesterol (mg/dL) | 17 | 16 | 17 | 13 |
| Apolipoprotein A-I (mg/dL) | 29 | 29 | 30 | 28 |
| Apolipoprotein A-II (mg/dL) | 2 | 5 | 6 | 5 |
| Apolipoprotein B (mg/dL) | 53 | 48 | 37 | 45 |
| Phospholipids (mg/dL) | 252 | 256 | 240 | 252 |
| Glucose (mg/dL) | 79 | 105 | 109 | 76 |
| *Kidney function* | | | | |
| eGFR (mL/min/1.73m$^2$) | 18.3 | 17.7 | 14.6 | 13.0 |
| Serum creatinine (mg/dL) | 3.7 | 3.8 | 4.4 | 4.8 |
| Albumin-to-creatinine ratio (mg/g) | 2496.9 | 3316.6 | 3097.9 | 2623.5 |
| Protein-to-creatinine ratio (mg/g) | 3037.8 | 4359.6 | 3564.1 | 3522.3 |
| Proteinuria (g/day) | 3.680 | 6.093 | 4.587 | 4.261 |
| *Complete blood count* | | | | |
| Red blood cells (x10$^6$/µL) | 3.28 | 3.29 | 3.69 | 3.20 |
| Haemoglobin (g/dL) | 9.7 | 9.6 | 10.5 | 9.2 |
| Haematocrit (%) | 29.3 | 29.0 | 32.1 | 28.0 |
| Platelets (x10$^3$/µL) | 150 | 154 | 105 | 121 |
| White blood cells (x10$^3$/µL) | 2.26 | 3.14 | 3.15 | 1.36 |
| Neutrophils (x10$^3$/µL) | 1.59 | 2.73 | 2.60 | 0.87 |
| Lymphocytes (x10$^3$/µL) | 0.38 | 0.22 | 0.36 | 0.34 |
| Monocytes (x10$^3$/µL) | 0.22 | 0.19 | 0.18 | 0.07 |
| Eosinophils (x10$^3$/µL) | 0.06 | 0.00 | 0.00 | 0.04 |

To convert the values for cholesterol to millimoles per liter, multiply by 0.02586. To convert the values for triglycerides to millimoles per liter, multiply by 0.01129. To convert the values for glucose to millimoles per liter, multiply by 0.05551. To convert the values for serum

creatinine to micromoles per liter, multiply by 0.011. The estimated glomerular filtration rate (eGFR) was calculated with the CKD-EPI equation. EOT denotes end of treatment, HDL high-density lipoprotein, LDL low-density lipoprotein.

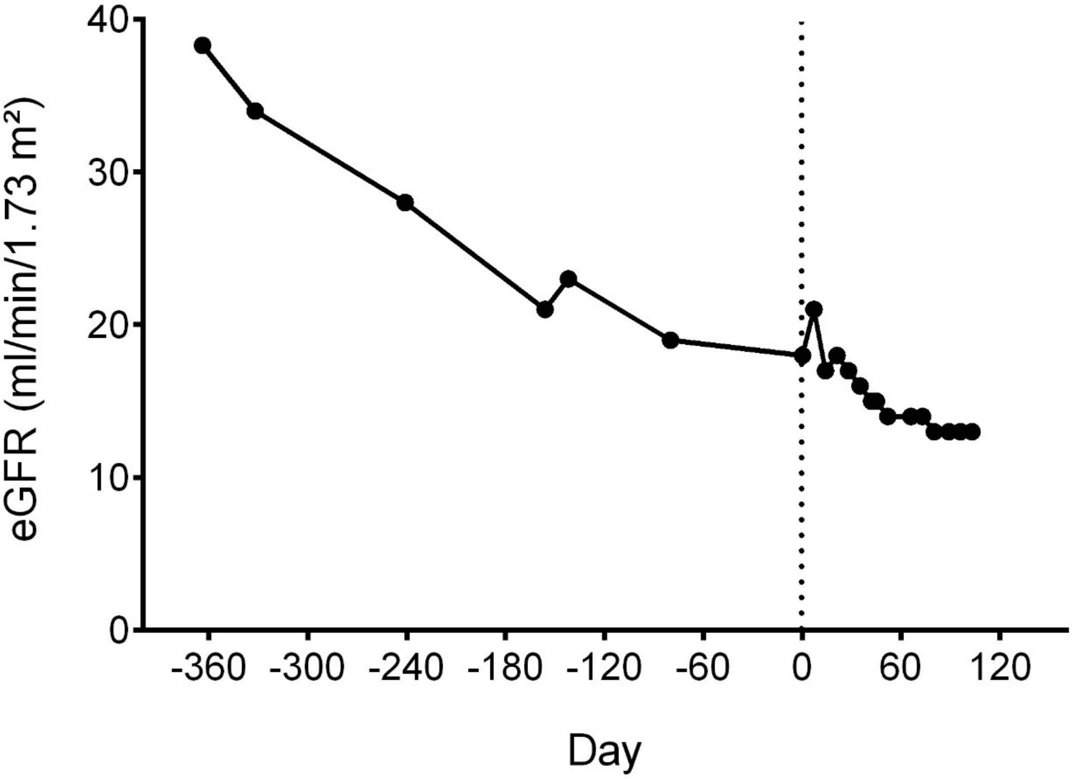


**Figure S1. Kidney function before and under CER-001 treatment.** Kidney function before and under CER-001 treatment divided by dotted line CER-001 administration. eGFR denotes estimated glomerular filtration rate.

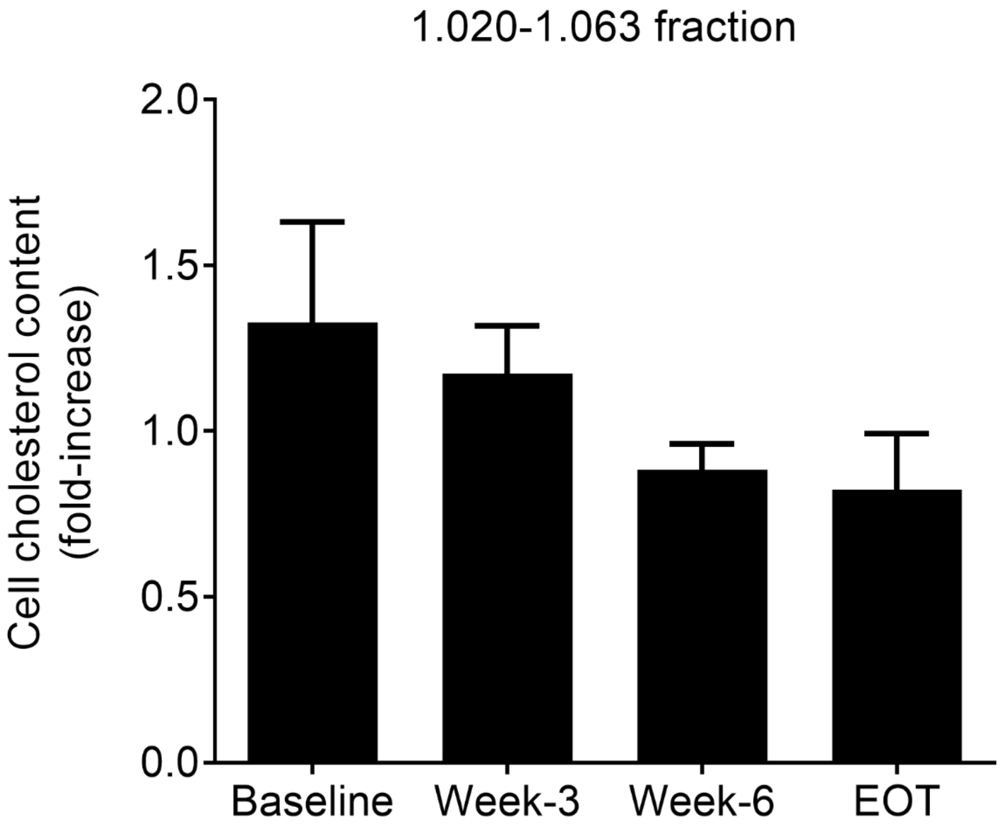


**Figure S2. Remodeling of plasma lipoproteins by CER-001 limits cholesterol deposition in cultured podocytes.** Podocytes were incubated with the 1.020-1.063 g/mL lipoprotein fraction isolated from patient's plasma collected at all time-points. Data are presented as mean ± SD. Data were analyzed by one-way ANOVA for repeated measures followed by Bonferroni's post hoc test. P-value vs baseline = 0.093.
